# Rapid identification of clinical common invasive fungi via a multi-channel real-time fluorescent polymerase chain reaction melting curve analysis

**DOI:** 10.1097/MD.0000000000019194

**Published:** 2020-02-14

**Authors:** Xiaozi Wen, Qiong Chen, Huali Yin, Shenghai Wu, Xianjun Wang

**Affiliations:** a Department of The Fourth Clinical Medical College affiliated to Zhejiang Chinese Medical University; b Department of Clinical Laboratory, Hangzhou First People's Hospital affiliated to Zhejiang Chinese Medical University; c Department of Research and Development, Hangzhou Qianji Biological Technology Co., Ltd., Hangzhou, China.

**Keywords:** candidemia, invasive fungal infection, melting curve analysis, real-time fluorescent polymerase chain reaction, melting temperature

## Abstract

The incidence of invasive fungal infections (IFIs) has recently increased, and early and accurate diagnosis of IFIs is important for the rational selection of antifungal drugs with high efficacy. We developed a method for rapid and accurate clinical diagnosis of IFIs and provide a reference for personalized drug treatment.

We designed and screened fungal internal transcribed spacer regions with universal primers and designed 8 TaqMan detection probes to establish a multi-channel real-time fluorescent polymerase chain reaction (PCR) melting curve analysis (MCA) method. The sensitivity, specificity, and reproducibility of this method were investigated using standard fungal strains and clinical isolates. Candidemia was detected using the MCA method.

The limit of detection and assay cut-off (melting temperature [Tm]) for *Candida albicans* were 0.05 pg/μL and 66.50 °C; *Candida glabrata* were 0.1 pg/μL and 66.25 °C; *Candida tropicalis* were 0.1 pg/μL and 60.15 °C; *Candida krusei* were 0.1 pg/μL and 72.15 °C; *Candida parapsilosis* were 0.2 pg/μL and 63.10 °C; *Candida guilliermondii* were 0.1 pg/μL and 61.84 °C; *Cryptococcus neoformans* were 0.1 pg/μL and 65.50 °C; *Aspergillus flavus* were 0.05 pg/μL and 71.50 °C; *Aspergillus terreus*, *Aspergillus fumigatus*, and *Aspergillus niger* were 0.05 pg/μL and 76.80 °C. Analytical specificity was evaluated using 13 clinical pathogens including *Streptococcus pneumoniae*, *Staphylococcus aureus*, and *Haemophilus influenzae*, etc. No false-positive results were obtained for any of these samples. The MCA method can detect and identify different candidemia simulations. The limit detection concentration of *C albicans* was 44 cfu/mL, *C glabrata* was 73 cfu/mL, *C tropicalis* was 29 cfu/mL, *C parapsilosis* was 21 cfu/mL, *C krusei* was 71 cfu/mL, and *C guilliermondii* was 53 cfu/mL.

The multi-channel real-time fluorescence PCR melting curve analysis displayed high sensitivity and specificity in detecting various clinically invasive fungi. Furthermore, it simultaneously detected the genera *Candida*, *Cryptococcus*, and *Aspergillus* and identified *Candida* at the species level. Our method can facilitate early and accurate clinical diagnosis and personalized medication regimens.

## 1 Introduction

Invasive fungal infections (IFIs) have increased with the widespread use of broad-spectrum antibiotics, immunosuppressive agents, antineoplastic drugs, and in-depth development of organ transplantation, and various invasive diagnostic techniques.^[1–3]^*Candida*, *Aspergillus*, *Pneumocystis*, and *Cryptococcus neoformans* are the primary pathogens causing IFIs, with *Candida* responsible for the largest number of cases,^[4,5]^ and the infection rates of *Aspergillus*, *Pneumocystis*, and *C neoformans* have increased.^[6]^ IFIs primarily occur in patients with severe underlying diseases, malignant tumors, and other severe diseases compromising immune function and in those undergoing organ transplantation.^[7]^ The clinical manifestations of IFIs are often non-specific and easily masked by primary underlying diseases. Early diagnosis is difficult, often resulting in delayed diagnosis, misdiagnosis, and delayed treatment. Furthermore, IFIs have a poor prognosis and are associated with high mortality,^[8]^ with approximately 1.4 million deaths worldwide each year.^[9]^ Sun et al^[10]^ reported that the total mortality rate with IFIs is 13.4%, and Barnes^[11]^ reported that the mortality rates associated with invasive candidiasis (IC) and invasive aspergillosis are 36% to 63% and 70%, respectively.

Laboratory detection of IFIs primarily involves traditional detection methods including direct microscopy, culture, and histopathology.^[12]^ Traditional methods have a low positive detection rate and poor sensitivity and do not yield a rapid positive clinical diagnosis.^[13]^ Serological analysis primarily includes the 1,3-β-D-glucan test (G test), galactomannan test (GM test), and latex agglutination test. Although these methods potentially provide a basis for the early diagnosis of IFIs, they can be influenced by various factors, leading to false-positive findings.^[14]^ Furthermore, they do not accurately detect fungal species.^[15]^ Molecular biology technologies based on PCR have been widely used to detect IFIs. Fluorescence PCR melting curve analysis (MCA) is an emerging detection method for identifying fungal species without sequencing.^[16]^ MCA has a high sensitivity, throughput, speed, and accuracy,^[17,18]^ and it is less expensive; thus, it is applicable for detecting fungal infections.^[19,20]^ This method is based on the principle that different double-stranded DNA molecules have different Tm values, and changes in the shape of the melting curve can be monitored using fluorescent dyes or probes to rapidly and accurately detect and identify various fungi.^[21]^ The TaqMan probe melting curve analysis uses the TaqMan probe rather than the SYBR Green dye to specifically bind to the target DNA. By analyzing the melting curve and Tm value of specific binding products, fungal detection at the species level can be achieved.^[22]^ Furthermore, TaqMan probes can link fluorescent reporter groups of different wavelengths, and thus completely harness the advantages of multi-channel fluorescent probe PCR for simultaneous detection of various fungi. For example, Alonso et al^[23]^ performed real-time fluorescent probe PCR melting curve analysis to detect and identify *Aspergillus*, and Valero et al^[24]^ also detected and identified *Mucor*, *Rhizopus*, and endemic diseased-related fungi and established a corresponding melting curve library. In this study, we used a combination of PCR amplification technology and multi-channel real-time fluorescent probe melting curve analysis for closed-tube detection and species identification of common IFI-causing pathogens (*Candida*, *Aspergillus*, and *Cryptococcus*). This study was conducted to develop a method for rapid and accurate clinical diagnosis of IFIs and provide a reference for personalized drug treatment.

## 2 Material and methods

### 2.1 Strain

The strains used in this study were subjected to sequencing analysis with specific primers (by Shanghai Sunny Biotech Co., Ltd., Shanghai, China). The following fungal strains were used (Table 1): *Candida albicans* (ATCC 10230), *Candida glabrata* (BNCC 337348), *Candida tropicalis* (BNCC 337310), *Candida parapsilosis* (BNCC 337317), *Candida krusei* (ATCC 6258), *Candida guilliermondii* (ATCC 6260), *Cryptococcus neoformans* (BNCC 337347), *Aspergillus flavus* (HB-CICC 71005), *Aspergillus terreus* (HB-CICC 70504), *Aspergillus niger* (01018094), and *Aspergillus fumigatus* (01010287). The following clinical isolates were used: *C albicans*, *C glabrata*, *C tropicalis*, *C parapsilosis*, *C krusei*, *C guilliermondii*, *Streptococcus pneumoniae*, *Streptococcus salivarius*, *Staphylococcus aureus*, *Staphylococcus epidermidis*, *Klebsiella pneumoniae*, *Haemophilus influenzae*, *Escherichia coli*, *Pseudomonas maltophilia*, *Pseudomonas aeruginosa*, *Nocardia amarae*, *Legionella* sp., *Bordetella pertussis*, and *Corynebacterium diphtheriae* (obtained from the First People's Hospital of Xiaoshan District, Hangzhou, China).

**Table 1:**
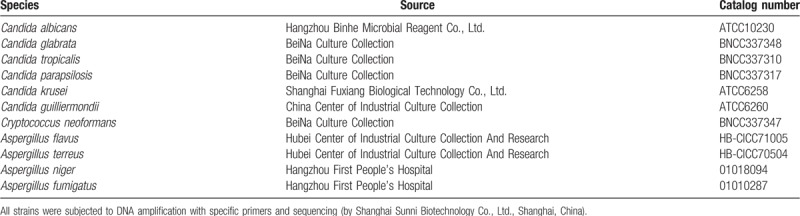
Fungal strains used in this study.

### 2.2 Blood samples

Blood was collected from patients who visited the First People's Hospital for a health checkup, with no clinical symptoms of sepsis and inflammatory markers.

### 2.3 DNA extraction

Strain preparation: Each fungal strain was inoculated on potato glucose liquid medium (Shanghai Qigong Biotechnology Co., Ltd., Shanghai, China) and cultured (30 °C, 4 × *g*, 2–3 days). Pretreatment: 200 μL aliquots of the culture was centrifuged (4 °C, 18,000 × *g*, 5 minutes), and then the pellet was treated with 600 μL of 1.2 M sorbitol and 10 U Lyticase (Tiangen Biochemical Technology Co., Ltd., Beijing, China), thoroughly mixed, incubated at 30 °C for 30 minutes to disrupt the fungal cell wall, and centrifuged (4 °C, 18,000 × *g*, 5 minutes), and the supernatant was discarded. DNA extraction: Genomic DNA of the fungi was extracted using the magnetic bead method with the fungal DNA extraction kit (Hangzhou Qianji Biological Technology Co., Ltd., Hangzhou, China) in accordance with the manufacturer's instructions. DNA purity and concentration were measured using the Nano-300 spectrophotometer (Hangzhou Aosheng Instrument Co., Ltd., Hangzhou, China). DNA samples were diluted to 1 ng/μL, 10, 1, 0.1, 0.05, and 0.01 pg/μL.

### 2.4 Design and synthesis of primers and probes

The internal transcribed spacer (ITS) in the fungal genome is a conserved, species-specific 18S rDNA–ITS1–5.8S rDNA–ITS2–28S rDNA tandem repeat. The ITS sequences of *C albicans* and *C glabrata*, *C tropicalis*, *C krusei*, *C parapsilosis*, *C guilliermondii*, *C neoformans*, *A flavus*, *A terreus*, *A niger*, and *A fumigatus* were compared and analyzed with the National Center for Biotechnology Information (NCBI) database. Based on the highly conserved/relatively specific segment of the ITS region of the strain, primer 5.0 software was used to design universal primers (ITSF1/ITSR1)/specific probes (CaP1/CgP1/CtP1/CkP1/CpP1/CguP1/CnP1/A. P1), and the probes were labeled with different fluorophores (Table 2).

**Table 2:**
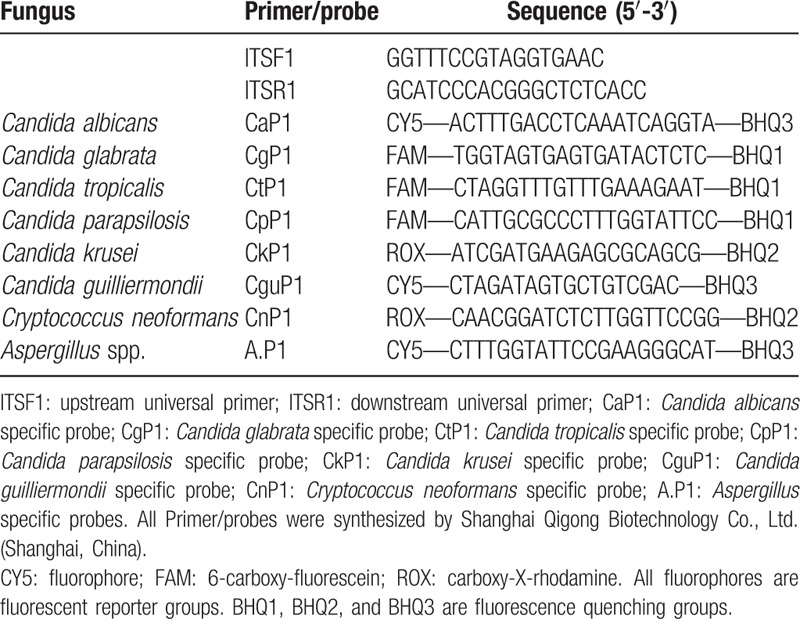
Primer and probe sequences.

### 2.5 PCR conditions and MCA

PCR amplification and MCA were performed using SLAN-96S (Shanghai Hongshi Medical Technology Co., Ltd., Shanghai, China). The PCR was carried out with a reaction mixture of volume 25 μL comprising 2.5 μL of 10× PCR buffer, 5 μL of 5× probe buffer, 0.2 mM dN(U)TPs, 1 U of Hot Start Taq polymerase, 0.2 U of UDG, 0.4 μM ITSF1, 0.04 μM ITSR1, 0.16 μM primers, and sterile water. The PCR program comprised a UDG enzyme reaction for 2 minutes at 50 °C, followed by initial denaturation for 3 minutes at 95 °C, 60 cycles for 30 seconds at 95 °C, 30 seconds at 56 °C, and 30 seconds at 72 °C, and termination for 2 minutes at 95 °C. The amplification products for MCA were cooled for 2 minutes at 40 °C and heated to 85 °C to monitor fluorescence at 0.06 °C/s. Thereafter, 1 ng/μL of DNA each reference fungal strain was analyzed in 10 independent runs and the mean melting temperature (Tm) and standard deviation (SD) values were determined for each melting domain.

### 2.6 Sensitivity and specificity

The genomic DNA samples of each fungal strain were diluted to 10, 1, 0.1, 0.05, and 0.01 pg/μL, and then 4 μL of each sample was analyzed in 20 independent runs and the limit of detection was determined. The genomic DNA of the clinical isolates was extracted using the DNA Extraction kit (Hangzhou Qianji Biological Technology Co., Ltd.) in accordance with the manufacturer's instructions, and 100 pg/μL of each sample was analyzed to evaluate the specificity of the analysis.

### 2.7 Detection of clinical sample

Candidemia’ clinical samples were detected using the MCA method. Clinical sample preparation: blood samples were drawn into a 2-mL anticoagulation tube (K2EDTA, BD Medical Equipment Co., Ltd. Shanghai, China). The 6 model clinical isolates of fungal strains were counted, and inoculated blood samples later, in order to obtain the fungal cell number of 10^3^, 10^2^, 10^1^ cfu/mL for each of simulated candidemia sample. The diluted sample (400 μL) was added into the pathogen lysis tubes (L) (QIAGEN, Germany) and treated with 10 U Lyticase, thoroughly mixed, and incubated at 30 °C for 30 minutes. DNA extraction: simulated candidemia samples’ DNA was extracted according to the manufacturer's instructions of the QIAmp UCP Pathogen kit (QIAGEN, Germany).

Furthermore, 10^6^ cfu/mL of each fungal suspension was used as the positive control and healthy human blood as the negative control. The MCA was performed with DNA samples from the positive control, negative control, and different concentrations of simulated candidemia samples. The research was approved by the local Bioethics Committee of Hangzhou First People's Hospital.

## 3 Results

### 3.1 MCA for reference strains

**Figure 1:**
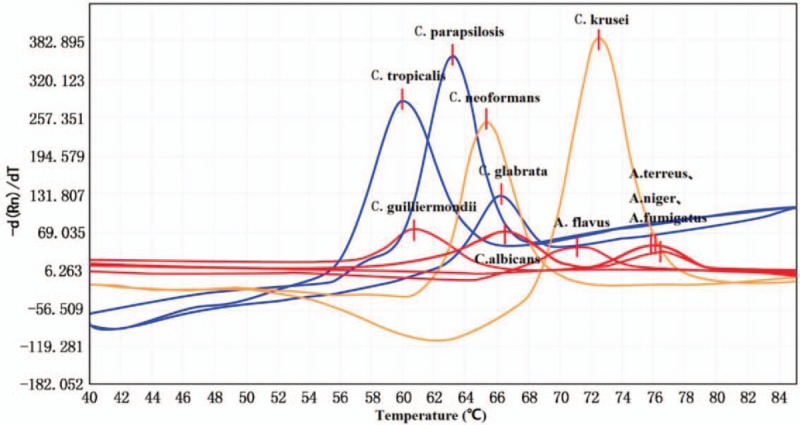
Fusion curves of 11 fungal strains with a single Tm value. *C albicans*: *Candida albicans*; *C glabrata*: *Candida glabrata*; *C tropicalis*: *Candida tropicalis*; *C parapsilosis*: *Candida parapsilosis*; *C krusei*: *Candida krusei*; *C guilliermondii*: *Candida guilliermondii*; *C neoformans*: *Cryptococcus neoformans*; *A flavus*: *Aspergillus flavus*; *A terreus*: *Aspergillus terreus*; *A niger*: *Aspergillus niger*; *A fumigatus*: *Aspergillus fumigatus*.

MCA using 6 *Candida* reference strains, 1 *Cryptococcus* reference strain, and 4 *Aspergillus* reference strains revealed reproducible melting peaks for each species. Most strains displayed a single peak (Fig. 1). All *Candida*, *Cryptococcus*, and *Aspergillus* species were directly identified by MCA. The mean Tm ± SD value(s) together with the shape of the melt curve distinguished the 6 *Candida* spp., 1 *Cryptococcus* sp., and 1 *Aspergillus* sp. (Fig. 1, Table 3). However, the other 3 *Aspergillus* spp. could not be reliably distinguished. The mean Tm ± SD values of *A terreus*, *A niger*, and *A fumigatus* were similar and overlapping.

**Table 3:**
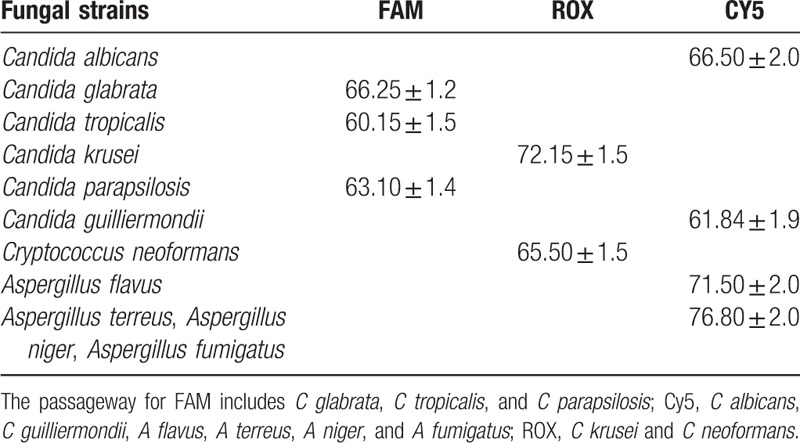
Tm ± SD values of fungal strains.

### 3.2 Sensitivity and specificity

**Figure 2:**
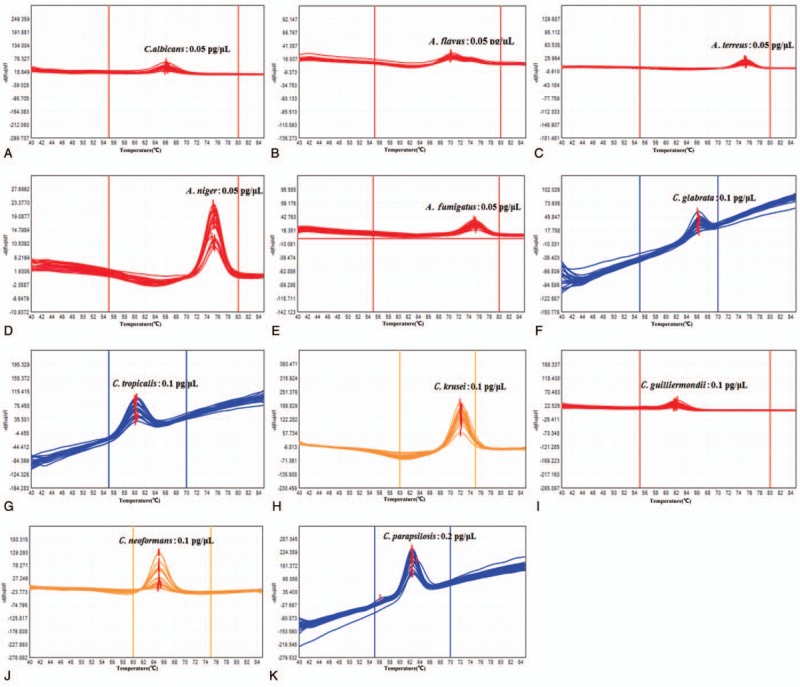
Sensitive detection of fungal strains. *C albicans*: *Candida albicans*; *C glabrata*: *Candida glabrata*; *C tropicalis*: *Candida tropicalis*; *C parapsilosis*: *Candida parapsilosis*; *C krusei*: *Candida krusei*; *C guilliermondii*: *Candida guilliermondii*; *C neoformans*: *Cryptococcus neoformans*; *A flavus*: *Aspergillus flavus*; *A terreus*: *Aspergillus terreus*; *A niger*: *Aspergillus niger*; *A fumigatus*: *Aspergillus fumigatus*. A: The limit of detection for *C albicans* was 0.05 pg/μL; B: the limit of detection for *A flavus* was 0.05 pg/μL; C: the limit of detection for *A terreus* was 0.05 pg/μL; D: the limit of detection for *A niger* was 0.05 pg/μL; E: the limit of detection for *A fumigatus* was 0.05 pg/μL; F: the limit of detection for *C glabrata* was 0.1 pg/μL; G: the limit of detection for *C tropicalis* was 0.1 pg/μL; H: the limit of detection for *C krusei* was 0.1 pg/μL; I: the limit of detection for *C guilliermondii* was 0.1 pg/μL; J: the limit of detection for *C neoformans* was 0.1 pg/μL; K: the limit of detection for *C parapsilosis* was 0.2 pg/μL.

**Figure 3:**
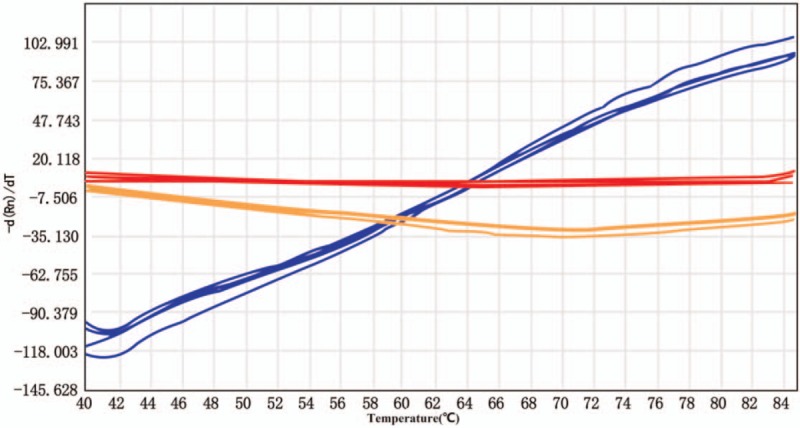
Specific detection of 13 common pathogens. The 13 common pathogens were *Streptococcus pneumoniae*, *Staphylococcus aureus*, *Klebsiella pneumoniae*, *Haemophilus influenzae*, *Escherichia coli*, *Pseudomonas maltophilia*, *Pseudomonas aeruginosa*, *Streptococcus salivarius*, *Staphylococcus epidermidis*, *Nocardia amarae*, *Legionella* sp., *Bordetella pertussis*, and *Corynebacterium diphtheriae*. None of them displayed a specific melting curve and Tm value.

The limit of detection for *C albicans*, *A flavus*, *A terreus*, *A niger*, and *A fumigatus* was 0.05 pg/μL; for *C glabrata*, *C tropicalis*, *C krusei*, *C guilliermondii*, and *C neoformans* was 0.1 pg/μL; and for *C parapsilosis* was 0.2 pg/μL. The detection rate of each fungal strain was >95% (Fig. 2). None of the 13 clinical bacterial isolates displayed specific melting curves and Tm values, and no cross-reaction with fungal detection was observed (Fig. 3).

### 3.3 Clinical sample

**Figure 4:**
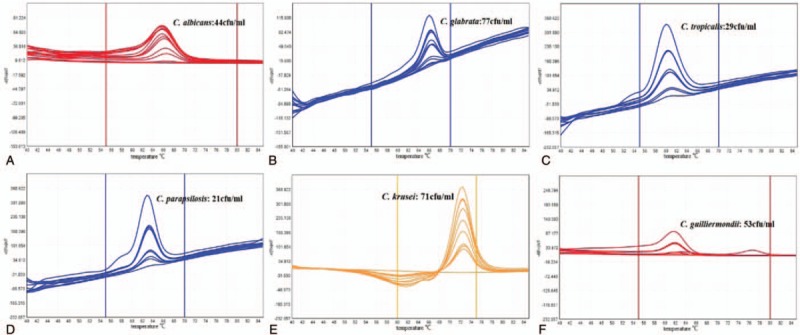
Fusion curves of 6 simulated candidemia samples. A: The lowest detection concentration of *C albicans* sample was 44 cfu/mL; B: the lowest detection concentration of *C glabrata* was 73 cfu/mL; C: the lowest detection concentration of *C tropicalis* was 29 cfu/mL; D: the lowest detection concentration of *C parapsilosis* was 21 cfu/mL; E: the lowest detection concentration of *C krusei* was 71 cfu/mL; F: the lowest detection concentration of *C guilliermondii* was 53 cfu/mL.

The positive control (10^6^ cfu/mL) displayed specific melting curve and Tm value, whereas the negative control did not show a specific response. The MCA method can detect simulated candidemia with different concentrations of fungal strain. The limit detection concentration of *C albicans* was 44 cfu/mL, *C glabrata* was 73 cfu/mL, *C tropicalis* was 29 cfu/mL, *C parapsilosis* was 21 cfu/mL, *C krusei* was 71 cfu/mL, and *C guilliermondii* was 53 cfu/mL (Fig. 4).

## 4 Discussion

As an emerging detection method, fluorescence PCR melting curve analysis is useful for diagnosing IFIs.^[25]^ In this study, for multi-channel real-time fluorescent PCR melting curve analysis, general ITS primers were first used to identify *Candida*, *Aspergillus*, and *Cryptococcus*, and then used specific TaqMan probes to obtain and analyze specific melting curves and Tm values for all fungi, which simultaneously distinguished *C albicans*, *C glabrata*, *C tropicalis*, *C krusei*, *C parapsilosis*, *C neoformans*, and *Aspergillus* spp. in the same reaction mixture. This method harnesses the advantages of multi-channel fluorescent probe PCR. Based on the reference Tm value of each fungal primer and probe sequence, the CaP1, CgP1, and CnP1 probes with similar Tm values were labeled with CY5, FAM, and ROX fluorophores, respectively; CtP1 and CguP1 with FAM and CY5; CkP1 and A.P1 with ROX and CY5; CpP1 with FAM. These probe-fluorophore pairs yielded specific, significantly different melting curves, and Tm values among fungi in their detection channels. This method involves an asymmetric PCR, that is, the amounts of the 2 primers are different, and when the restriction primers at relatively low concentrations are completely utilized, the other unrestricted primers are amplified, yielding the target single-stranded DNA, which pairs and binds with specific probes. As the temperature of the PCR system increases, target genes can be identified based on the melting curve probe peak. Asymmetric PCRs are influenced by primer concentration and ratio, probe concentration, and Mg^2+^ concentration,^[26]^ and the annealing temperature is critical for successful asymmetric PCR. The PCR system for multi-channel real-time fluorescence PCR melting curve analysis was 25 μL, and the primer concentration was gradually increased from 0.04 to 0.4 μM, the ratio of upstream and downstream primers from 5:1 to 20:1, the probe concentration from 0.04 to 0.24 μM, and dN(U)TPs from 0.20 to 0.24 μM, and the annealing temperature from 50 to 60 °C to optimize the system and finally determine the optimal PCR system and conditions.

The multi-channel real-time fluorescence PCR melting curve analysis is a closed system, thus effectively preventing false-positive results caused by external influences. This method has high sensitivity and specificity and displays a lower limit of detection of 0.5 to 1 pg/mL during MCA than the method devised by Nemcova et al,^[27]^ with the former showing higher sensitivity. During fungal identification, based on specific melting curves and Tm values, this method distinguished *C albicans*, *C glabrata*, *C tropicalis*, *C krusei*, *C parapsilosis*, *C neoformans*, and *A flavus*. However, it was difficult to distinguish and identify *A fumigatus*, *A terreus*, and *A niger* using this method because of the similarity of their melting curves and Tm values, which was likely related to the specificity of the A.P1 probe. With respect to the entire probe for >25 nucleotides, a certain degree of conservation among fungal genera and species specificity is required, which would adjust the probe position and Tm values for interaction with the appropriate probe sequence, such that it can be completely combined with the corresponding sequences and simultaneously distinguish fungi. Furthermore, the development of a single probe with greater specificity to identify four *Aspergillus* spp. is even more challenging. The A.P1 probe detects *Aspergillus* at the genus level; however, it cannot identify *A fumigatus*, *A terreus*, and *A niger*. This issue can be resolved by designing and synthesizing a single *A fumigatus* probe, *A flavus* probe, *A terreus* probe, and *A niger* probe to rapidly identify clinically common *Aspergillus* spp.

Invasive candidiasis (IC) is an important type of IFIs, and it has become a major threat to public health.^[28]^ To date, more than 17 different *Candida* species have been identified as pathogens of IC, and *C albicans* is the leading and most widely studied pathogen.^[29]^ However, in recent years, the proportion of *non-C albicans* has increased rapidly.^[30]^ The antibacterial activity of antibacterial drugs varies with strains, and the choice of treatment drugs is also different^[31]^; early identification of the pathogenic species of IC is essential for early clinical medication. In this study, we detected IC using the MCA method; 6 clinically isolated fungal strains were used to simulate candidemia samples, which included 6 positive controls, 1 negative control, and 3 concentration gradients of candidemia samples, and then the MCA was performed. All positive controls were positive, whereas the negative control showed no reaction. The results showed that there was no positive effect of healthy human blood on the established MCA detection system, and each clinical isolate of *Candida* strain (10^6^ cfu/mL) could be detected successfully. Furthermore, the MCA method can detect simulated candidemia with different concentrations of fungal strains, and the lowest detection concentration of *C glabrata* and *C krusei* was 70 to 80 cfu/mL, *C albicans* and *C guilliermondii* was 40 to 50 cfu/mL, *C tropicalis* and *C parapsilosis* was 20 to 30 cfu/mL (the concentrations were calculated from the results of the diluted coated plate count). However, there was a melting curve peak of *C parapsilosis* blood sample at Tm of around 76 °C, which was that of *Aspergillus*. That is, there was contamination by *Aspergillus* in the detection process. Therefore, the test results of *C parapsilosis* should be deliberated.

In summary, compared with the gold standard culture method, the MCA method can detect candidemia samples and yield results within 4 to 6 hours, and its sensitivity was 20 to 80 cfu/mL, indicating that the method is fast, accurate, and sensitive. However, our study had some limitations. The clinical sample is a simulated candidemia sample, and not a clinical sample, and we plan to collect samples from patients with invasive candidiasis for verification. Multi-channel real-time fluorescence PCR melting curve analysis is rapid and accurate compared with the traditional culture methods. This method has a high sensitivity and specificity and can simultaneously detect various common invasive fungi, invasive candidiasis especially, yielding detection results within 4 to 6 hours and facilitating early diagnosis of clinical IFIs. In the future, multi-channel real-time fluorescent PCR melting curve analysis may be combined with other detection methods (G and GM test), to potentially improve the early diagnosis rate of IFIs and providing a reference for clinical rationalization of antifungal treatment, reducing misdiagnosis, improving prognosis, and reducing mortality during clinical diagnosis and treatment.

## Author contributions

**Conceptualization:** Xianjun Wang, Qiong Chen, Huali Yin, Xiaozi Wen, Shenghai Wu.

**Data curation:** Xiaozi Wen, Qiong Chen, Huali Yin, Xianjun Wang.

**Formal analysis:** Xiaozi Wen, Qiong Chen, Huali Yin, Xianjun Wang.

**Funding acquisition:** Xianjun Wang

**Investigation:** Xiaozi Wen, Qiong Chen, Huali Yin, Xianjun Wang, Shenghai Wu.

**Methodology:** Xiaozi Wen, Qiong Chen, Huali Yin, Xianjun Wang.

**Project administration:** Qiong Chen, Huali Yin, Xiaozi Wen.

**Resources:** Qiong Chen, Huali Yin, Xianjun Wang, Shenghai Wu.

**Software:** Qiong Chen, Huali Yin, Xianjun Wang, Shenghai Wu.

**Supervision:** Qiong Chen, Huali Yin, Xianjun Wang.

**Validation:** Xiaozi Wen, Qiong Chen, Huali Yin, Xianjun Wang.

**Visualization:** Xiaozi Wen, Qiong Chen, Huali Yin, Xianjun Wang.

**Writing – original draft:** Xiaozi Wen, Qiong Chen, Huali Yin, Xianjun Wang.

**Writing – review & editing:** Xiaozi Wen, Qiong Chen, Xianjun Wang.
